# Relationship between serum 25 hydroxyvitamin D (25(OH)D) levels and mental health in shift female nurses

**DOI:** 10.1038/s41598-022-18721-8

**Published:** 2022-08-26

**Authors:** Hsin-Ya Tang, Wang-Sheng Ko, Yuan-Horng Yan, Su-Chen Yu, Ya-ling Chiou

**Affiliations:** 1 Department of Nutrition, Master of Biomedical Nutrition Program, Hungkuang University, No. 1018, Sec. 6, Taiwan Boulevard, Shalu District, 433304 Taichung, Taiwan, Republic of China; 2 Department of Nursing, Kuang-Tien General Hospital, Taichung, Taiwan; 3 Department of Internal Medicine, Kuang-Tien General Hospital, Taichung, Taiwan; 4 Department of Medicine Research, Kuang-Tien General Hospital, Taichung, Taiwan

**Keywords:** Health occupations, Risk factors

## Abstract

The nurses work long hours and in various shifts, and often accompanied by depression, fatigue, and sleep disorders. Many studies have found that 25 hydroxyvitamin D (25(OH)D) is related to mental health. We aimed to investigate the relationship between depression, sleep problems, fatigue, and serum 25(OH)D levels in shift nurses. We recruited 34 day-shift, 30 evening-shift and 31 night-shift nurses. The Beck Depression Inventory II (BDI-II), Numerical Rating Scale and General Sleep Disturbance Scale to evaluate the levels of depression, sleep problems, fatigue. Blood samples (20 ml) were collected under a fasting state to determine basic biochemistry and inflammatory parameters. In central of Taiwan, approximately 96.1% of shift nurses had deficient (< 20 ng/ml) (45 females and 1 male) and inadequate (20–29 ng/ml) (39 females and 2 male) 25(OH)D levels. Approximately 84.2% of shift nurses experienced fatigue. In sleep disturbance, night-shift nurses experienced significantly more severe sleep disturbance than day-shift and evening-shift nurses. However, no significant correlation was observed between 25(OH)D levels and mental health when the 25(OH)D level was categorized. 25(OH)D deficiency, sleep disturbance, depression, and fatigue were common in shift female nurses, but it was not possible to demonstrate the impact of 25(OH)D deficiency on the mental health of shift nurses in Taiwan.

## Introduction

It is important to promote the mental health of shift nurse staff. Many studies indicates that shift nurse often experience the following problems: (a) circadian rhythms, because sleep-wake cycle disturbances, resulting in poor sleep and/or increased daytime sleepiness, and sleep disorders increase (b) depression and (c) fatigue disorder^1–4^. Akerstedt et al.^5^ pointed out that the health problems most frequently complained by shift workers are sleep disorders and unrecoverable fatigue, and 80.2% of nurses have poor sleep quality^6^. In Asia, 38–65% of hospital nurses have depression disorder^7–10^. The effect of shift work on the physical and mental health of nursing staff cannot be ignored. Most nurses working on hospital wards are locked into three-shift work arrangements, and recent findings suggest an association between shift work sleep disturbances and work accidents, reduced job performance and job satisfaction, distress, cognitive deficits. The lack of adequate sleep is the main reason for leaving work to increase turnover intention by 4.60 times^11^. The high turnover rate of nursing staff in hospitals seriously affects the quality of care.

Physiological circadian rhythms problems often encountered by shift nurses are often the biggest factors affecting personal health. Many studies have long confirmed that working at night increases the risk of melatonin secretion disorder, which indirectly leads to breast cancer and rectal cancer^12–15^. Shift work easily leads to mental health problems. Hence, improving the mental health of shift workers should be taken into consideration.

Several studies have investigated the prevalence of sleep problems, stress, and fatigue related with the concentration of tumour necrosis factor alpha (TNF-α), and interleukin 6 (IL-6)^16^. The concentration of 1,25(OH)2D_3_ is also an important indicator.1,25(OH)_2_D_3_ has gradually gained research attention in recent years. It is not only a vitamin but also a hormone that is associated with many diseases such as neurodegenerative diseases, cardiovascular diseases, chronic inflammatory diseases, cancer, diabetes, and autoimmunity. Some studies found that the 1,25(OH)_2_D_3_ receptor is present in many organs of the body^17^. 1,25(OH)_2_D_3_ binds to its receptors and regulates the cell cycle, thus affecting organ function. The level of 25(OH)D was also negatively correlated with the incidence of colorectal and breast cancers^18^. This evidence suggests that 25(OH)D plays a protective role against various diseases. In other respects, many studies have pointed out that low 25(OH)D levels increase the incidence of depression^19,20^, while higher 25(OH)D levels can reduce the risk of depression^21^. Previous studies conducted in older adults and young women indicated that the levels of 25(OH)D can be used as an indicator of depression^22,23^. The lack of 25(OH)D, which increases the inflammatory response, may be related to the occurrence of depression^24,25^. Some studies indicated that women with low levels of 25(OH)D have higher serum IL-6 levels, and supplementation with 25(OH)D can reduce the levels of inflammatory factors^26^. This study aimed to explore the relationship between the levels of 25(OH)D and depression in shift nurses, tendency to determine whether 25(OH)D deficiency can be used as an indicator for predicting the risk of mental health disorder and to develop strategies for improving the physical and mental health of shift workers in the future.

## Methods

### Recruitment of participants

**Figure 1:**
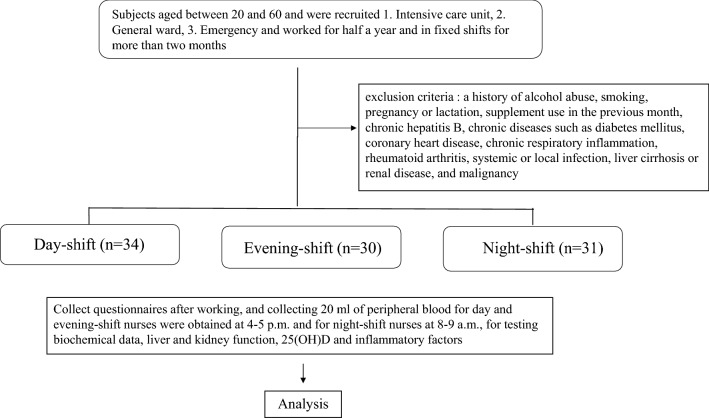
Flowchart for collecting subjects.

This study is a prospective study. Total of 95 volunteer nurses working in Kuang-Tien General Hospital (Taichung County, Taiwan) in 2016 were recruited in the study (Fig. 1). This study was approved by the Ethics in Human Research Committee (no. 10503) from Institutional Review Board of Kuang-Tien General Hospital, and all participants signed an informed consent prior to their inclusion in the study. The written informed consent was obtained from all subjects and/or their legal guardian(s). This study was registered retrospectively, and registration number TCTR20220623002 and the date of registration was 23/06/2022. Participants who had a history of alcohol abuse, smoking, pregnancy or lactation, supplement (including 25(OH)D) use in the previous month, chronic hepatitis B, chronic diseases such as diabetes mellitus, coronary heart disease, chronic respiratory inflammation, rheumatoid arthritis, systemic or local infection, liver cirrhosis or renal disease, and malignancy were excluded. A total of 95 volunteer nurses from the general ward, intensive care unit, and emergency room were included in the final analysis. These nurses have worked more than half a year and were assigned on a fixed shift (day, night, and evening shift) for more than 2 months. Of the 95 volunteer nurses, 34 were day-shift, 30 were evening-shift, and 31 were night-shift nurses. Each participant completed a questionnaire survey using a basic information questionnaire, Beck Depression Inventory II (BDI-II) questionnaire, Numerical Rating Scale for Fatigue (NRS-F), and General Sleep Disturbance Scale (GSDS); blood samples were collected from each participant after a 3-day duty.

### Questionnaire

Four types of questionnaires were used in this study. The basic information questionnaire is used to collect more general information such as patient’s name, height and weight, age, education level, marital status, work units, shifts (day, night, and graveyard shift), and the cumulative number of months in this shift. The BDI-II—Chinese version is used to measure the severity of depressive symptoms: < 10 points, normal; 10–18 points, mild depression; 19–29 points, moderate depression; 30–63 points, severe depression; and higher scores, more severe degree of depression^27^. The NRS-F is a seven-item questionnaire^28^, with each item rated from 0 (no fatigue) to 10 (very tired). This scale is used to rate the level of fatigue. Higher total scores and average scores indicate a more severe fatigue. An average score of 3.3 points or higher indicates a high level of fatigue. A previous Chinese study reported a Cronbach’s α reliability coefficient of 0.87–0.97. The GSDS is a 15-item questionnaire used to rate the frequency of specific sleep problems in the last 7 days^28^. Higher total and average scores indicate more sleep disorders. An average score of 3 or higher indicates clinically significant sleep disorder. The validity of the GSDS questionnaire was evaluated in 760 female shift nurses using the modified Stanford Sleep Questionnaire. The overall Cronbach's α coefficient was 0.88.

### Blood sample collection

Blood samples (20 ml) were collected under a fasting state. The serum was then separated from the cells by centrifugation, and the samples were stored at – 80 °C until analysis. The serum levels of total cholesterol, triglyceride, albumin, white blood cells (WBCs), lymphocytes, monocytes, neutrophils, eosinophils, basophils, total iron binding capacity (TIBC), red blood cells (RBC), platelets, hemoglobin (Hb) and 25(OH)D were measured by using the standard procedures of a hospital laboratory.

### Determining inflammatory parameters

The serum levels of AST, ALT, hs-CRP, and uric acid were measured using an automated clinical chemistry analyzer (FUJI DRI-CHEM 4000i). The serum levels of ferritin, IL-8, IFN-γ, IL-6, and TNF-α^16,29,30^ were measured using enzyme-linked immunosorbent assay (Biosense Laboratories, Norway). Ferritin levels were measured using a human ferritin enzyme immunoassay test kit (IBL-Hamburg kit, Germany) according to the manufacturer’s instructions.

All methods were performed in accordance with the relevant guidelines and regulations.

### Statistical analysis

Statistical analysis was performed using the Chinese version of SPSS version 22 (IBM SPSS statistics). Continuous variables with a normal distribution were analysed using analysis of variance. Values were expressed as mean ± standard deviation. For variables with non-normal distribution, the Kruskal–Wallis test was used. Categorical variables were analysed using a chi-square test. If the total number of fines in each of the fines less than five is more than 20%, the Fisher’s exact test was used. Values were expressed as percentages (%). *p* value < 0.05 was considered significant. G*Power software provides statistical methods. A post-hoc analysis is typically conducted after the completion of the study. As the sample size N is given, effect size is 0.33, Alpha = 0.05, the power (1−β) = 0.80 is calculated using the given N, the effect size, and the desired α level.

### Institutional Review Board statement

This study was approved by the Ethics in Human Research Committee (no. 10503). This study was registered retrospectively, and registration number TCTR20220623002 and the date of registration was 23/06/2022.

## Results

### Clinical characteristics

The characteristics and clinical data of shift nurses are shown in Tables 1 and 2. A total of 95 shift nurses were included in the study (day shift, 34; evening shift, 30; and night shift, 31). The day-shift nurses were significantly older than the evening-shift and night-shift nurses (*p* < 0.001). In terms of marital status, the proportion of single participants assigned in the evening shift (90%) and night shift (90.4%) was significantly greater than that in the day shift (26.5%) (Tables 1). Meanwhile, a significant difference was observed in the only WBC count between day-shift nurses and evening-shift nurses (Tables 2).

**Table 1:** Participant characteristics of three shift Nursing Staffs.

Variables	Shift			*p*-value
	Day (n = 34)	Evening (n = 30)	Night (n = 31)	
**Gender**				
Female	32 (94.1%)	30 (100%)	30 (96.8%)	0.772
Male	2 (5.9%)	0 (0.0%)	1 (3.2%)	
Age	36.5 (31.8–39.3)	25 (23.0–28.5)	25 (23.0–30.0)	< 0.001*^§￥^
BMI (kg/m^2^)	21.9 (20.2–23.5)	19.9 (18.4–22.4)	21.1 (19.4–23.4)	0.183
**Marital status**				
Single	9 (26.5%)	27 (90%)	28 (90.3%)	< 0.001*
Married	25 (73.5%)	3 (10.0%)	3 (9.7%)	
**Current unit**				
Ward	21 (61.8%)	20 (66.7%)	14 (45.2%)	0.702
MICU	4 (11.8%)	2 (6.7%)	4 (12.9%)	
SICU	7 (20.6%)	7 (23.3%)	10 (32.3%)	
ER	2 (5.9%)	1 (3.3%)	3 (9.7%)	
**Years of work in this unit**				
Less than half a year	5 (14.7%)	19 (63.3%)	12 (38.7%)	< 0.001*^c^
Seven months to one year	2 (5.9%)	1 (3.3%)	6 (19.4%)	
More than a year	27 (79.4%)	10 (33.3%)	13 (41.9%)	
**Education level**				
Five-year junior college	7 (20.6%)	10 (33.3%)	9 (29.0%)	0.733^c^
Two-year college of technology	8 (23.5%)	4 (13.3%)	5 (16.1%)	
University or above	19 (55.9%)	16 (53.3%)	17 (54.8%)	

BMI, body mass index; MICU, Medical Intensive Care Unit; SICU, surgical intensive care unit; ER, emergency room.

*p-value < 0.05(^§^Day shift *v.s.* evening shift; ^₤^ evening shift *v.s.* night shift; ^￥^Day shift *v.s.* night shift). The power after calculation and analysis after the event: the number of subjects is 95 (n = 95), and the ANOVA test is carried out in three groups, and the calculated power is 0.6550386.

^a^Data were continuous and presented as mean ± SD using ANOVA, The Tukey–Kramer test was performed for the post hoc multiple of ANOVA;

^b^Data were continuous and presented as median (IQR) using Kruskal–Wallis test, The Dunn test was performed for the post hoc multiple of Kruskal–Wallis test;

^c^Data were categorical and presented as n (%) using Fisher's exact test;

^d^Data were categorical and presented as n (%) using chi-squared test.

**Table 2:** Participant biochemistry of three class shift nurses.

Variables	Shifts			*p*
	Day (n = 34)	Evening (n = 30)	Night (n = 31)	
WBC (10^3^/μl)	5.3 (4.6–6.2)	7.3 (6.2–8.6)	6.0 (5.3–8.0)	< 0.001*^§b^
RBC (10^3^/μl)	4.7 (4.5–5.0)	4.8 (4.5–4.9)	4.7 (4.4–5.1)	0.995^b^
Hb (gm %)	13.5 (12.6–14.0)	13.5 (12.8–14.1)	13.4 (12.4–14.4)	0.707^b^
Platelet (1000/μl)	292.4 ± 78.6	307.8 ± 65.5	282.9 ± 67.8	0.391^a^
Triglyceride (mg/dl)	74.0 (47.0–96.8)	71.0 (55.5–92.3)	69.0 (54.0–94.0)	0.920^b^
Total Cholesterol (mg/dl)	173.0 (145.8–194.3)	176.0 (165.2–214.8)	184.0 (166.0–196.0)	0.098^b^
AST (U/l)	17.5 (15.8–22.0)	17.0 (15.0–21.0)	17.0 (15.0–19.0)	0.716^b^
ALT (U/l)	12.0 (9.0–16.0)	10.5 (8.8–16.0)	14.0 (11.0–15.0)	0.172^b^
Albumin (mg/dl)	4.6 (4.5–4.8)	4.8 (4.5–4.9)	4.7 (4.5–4.9)	0.444^b^
CRP (mg/dl)	0.0 (0.0–0.1)	0.0 (0.0–0.1)	0.0 (0.0–0.1)	0.575^b^
Ferritin (ng/ml)	43.3 (17.2–76.9)	49.5 (25.0–95.1)	39.4 (23.8–62.7)	0.684^b^
serum iron (μg/dl)	77.5 (63.0–123.3)	94.0 (60.8–122.3)	87.0 (63.0–106.0)	0.683^b^
TIBC (μg/dl)	319.0 (285.0–363.5)	311.0 (294.0–355.5)	317.0 (282.0–380.0)	0.853^b^
25(OH)D (ng/ml)	21.1 ± 6.2	21.3 ± 6.9	20.1 ± 4.7	0.730^a^
IL-6 (pg/ml)	0.0 (0.0–0.0)	0.0 (0.0–0.0)	0.0 (0.0–0.0)	0.966^b^
TNF-α (pg/ml)	0.1 (0.0–10.4)	3.3 (0.0–13.7)	7.9 (0.2–21.1)	0.051^b^
IL-8 (pg/ml)	1.4 (0.8–2.1)	1.2 (0.7–2.2)	1.9 (0.9–2.8)	0.394^b^
IFN-γ (pg/ml)	2.6 (0.0–10.8)	4.8 (0.2–18.9)	4.4 (0.8–19.2)	0.679^b^

WBC, white blood cell count; RBC, red blood cells; Hb, hemoglobin; AST, aspartate aminotransferase; ALT, alanine aminotransferase; CRP, C-Reactive Protein; TIBC, total iron binding capacity; IL-6, interleukin-6; TNF-α, tumor necrosis factor-α; IL-8, interleukin-8; IFN-γ, interferon-γ.

*p-value < 0.05(^§^Day shift *v.s.* evening shift; ^₤^ evening shift *v.s.* night shift; ^￥^Day shift *v.s.* night shift). The power after calculation and analysis after the event: the number of subjects is 95 (n = 95), and the ANOVA test is carried out in three groups, and the calculated power is 0.6550386.

^a^Data were continuous and presented as mean ± SD using ANOVA, The Tukey–Kramer test was performed for the post hoc multiple of ANOVA.

^b^Data were continuous and presented as median (IQR) using Kruskal–Wallis test, The Dunn test was performed for the post hoc multiple of Kruskal–Wallis test.

### Correlation between inflammatory data and 25(OH)D in all shift nurses groups

After dividing the 25(OH)D levels into < 20 ng/ml (n = 46), 20–29 ng/ml (n = 41), and ≥ 30 ng/ml (n = 8), no significant difference was observed in the levels of 25(OH)D among the shift nurses from all three categories using ANOVA method (Tables 3). However, 48.4% and 43.2% of shift nurses have severe and insufficient of 25(OH)D. No significant difference was also observed in the levels of inflammatory markers, including hs-CRP, IL-6, TNF-α, IL-8, and IFN-γ (Table 4).

**Table 3:** The characteristics of three class shift nurses according to 25(OH)D_3_ concentrations.

Variables	Serm 25(OH)D_3_, ng/ml			*p*
	< 20 (n = 46)	20–29 (n = 41)	≧30 (n = 8)	
**Shift**				
Day	15(32.6%)	16(39.0%)	3(37.5%)	0.186^c^
Evening	13(28.3%)	12(29.3%)	5(62.5%)	
Night	18(39.1%)	13(31.7%)	0(0.0%)	
**Gender**				
Female	45(97.8%)	39(95.1%)	8(100.0%)	0.693^c^
Male	1(2.2%)	2(4.9%)	0(0.0%)	
Age	28.0(23.0–36.0)	29.0(24.0–36.5)	28.0(34.3–31.5)	0.532^b^
BMI (kg/m^2^)	21.1(19.4–22.9)	21.1(19.7–25.5)	19.3(18.4–20.1)	0.133^b^
**Marital status**				
Single	33(71.7%)	26(63.4%)	5(62.5%)	0.678^d^
Married	13(28.3%)	15(36.6%)	3(37.5%)	
**Current unit**				
Ward	26(56.5%)	24(58.5%)	5(62.5%)	0.996^c^
MICU	5(10.9%)	4(9.8%)	1(12.5%)	
SUCU	11(23.9%)	11(26.8%)	2(25.0%)	
ER	4(8.7%)	2(4.9%)	0(0.0%)	
**Years of work in this unit**				
Less than half a year	18(39.1%)	14(34.1%)	4(50.0%)	0.081^c^
Seven months to one year	1(2.2%)	8(19.5%)	0(0.0%)	
More than a year	27(58.7%)	19(46.3%)	4(50.0%)	
**Education level**				
Five-year junior college	14(30.4%)	8(19.5%)	4(50.0%)	0.652^c^
Two-year college of technology	12(26.1%)	5(12.2%)	0(0.0%)	
University or above	20(43.5%)	28(68.3%)	4(50.0%)	

BMI, body mass index; MICU, Medical Intensive Care Unit; SICU, surgical intensive care unit; ER, emergency room.

*p-value < 0.05. The power after calculation and analysis after the event: the number of subjects is 95 (n = 95), and the ANOVA test is carried out in three groups, and the calculated power is 0.6550386.

^a^Data were continuous and presented as mean ± SD using ANOVA.

^b^Data were continuous and presented as median (IQR) using Kruskal–Wallis test.

^c^Data were categorical and presented as n (%) using Fisher's exact test.

^d^Data were categorical and presented as n (%) using chi-squared test.

**Table 4:** Participant biochemistry according to 25(OH)D_3_ concentrations.

Variables	Serum 25(OH)D_3_, ng/ml			*p*
	< 20 (n = 46)	20–29 (n = 41)	≧ 30 (n = 8)	
WBC (10^3^/μl)	6.2 ± 1.6	6.8 ± 1.9	6.3 ± 1.5	0.303^a^
RBC (10^3^/μl)	4.7 (4.4–4.9)	4.8 (4.5–5.0)	4.7 (4.5–5.0)	0.132^b^
Hb (gm %)	13.2 (12.4–14.1)	13.6 (12.8–14.2)	13.6 (12.9–13.8)	0.389^b^
Platelet (1000/μl)	289.0 ± 68.3	295.3 ± 77.5	318.3 ± 52.6	0.561^a^
Triglyceride (mg/dl)	69.0 (51.0–92.5)	76.0 (54.5–97.5)	63.5 (53.0–86.5)	0.624^b^
Total Cholesterol (mg/dl)	183.1 ± 27.3	180.5 ± 30.0	170.1 ± 32.5	0.505^a^
AST (U/L)	17.0 (16.0–21.0)	17.0 (15.0–19.0)	18.5 (15.3–24.8)	0.541^b^
ALT (U/L)	13.0 (9.8–16.5)	13.0 (10.0–15.5)	9.5 (8.3–21.0)	0.475^b^
Albumin(mg/dl)	4.7 ± 0.3	4.7 ± 0.3	4.8 ± 0.3	0.414^a^
CRP (mg/dl)	0.0 (0.0–0.1)	0.0 (0.0–0.1)	0.1 (0.0–0.1)	0.602^b^
Ferritin (ng/ml)	34.5 (20.7–62.9)	52.3 (25.4–73.3)	66.9 (20.8–130.3)	0.230^b^
serum iron (μg/dl)	94.2 ± 40.1	86.7 ± 36.7	100.4 ± 47.5	0.539^b^
TIBC (μg/dl)	319.5 (292.5–365.0)	315.0 (283.5–364.5)	315.0 (293.0–340.0)	0.960^a^
IL-6 (pg/ml)	0.0 (0.0–0.0)	0.0 (0.0–0.0)	0.0 (0.0–0.0)	0.429^b^
TNF-α (pg/ml)	3.0 (0.0–17.5)	2.6 (0.0–14.9)	4.8 (0.0–15.6)	0.957^b^
IL-8 (pg/ml)	1.4 (0.8–2.8)	1.3 (0.7–2.0)	1.6 (0.6–3.4)	0.748^b^
IFN-γ (pg/ml)	2.3 (0.3–15.3)	4.5 (0.1–14.5)	6.4 (1.3–16.0)	0.757^b^

WBC, white blood cell count; RBC, red blood cells; Hb, hemoglobin; AST, aspartate aminotransferase; ALT, alanine aminotransferase; CRP, C-Reactive Protein; TIBC, total iron binding capacity; IL-6, Interleukin-6; TNF-α, tumor necrosis factor-α; IL-8, interleukin-8; IFN-γ, interferon-γ.

*p-value < 0.05. The power after calculation and analysis after the event: the number of subjects is 95 (n = 95), and the ANOVA test is carried out in three groups, and the calculated power is 0.6550386.

^a^Data were continuous and presented as mean ± SD using ANOVA.

^b^Data were continuous and presented as median (IQR) using Kruskal–Wallis test.

### Correlation between questionnaire scores and 25(OH)D

We used three types of questionnaires (Beck BDI-II, NRS-F, and GSDS) to obtain and analyze the data of all nurses. Night-shift nurses obtained higher total and average GSDS scores than evening-shift and day-shift nurses significantly (Table 5). After dividing the 25(OH)D levels into three groups, the scores of the three questionnaires were compared and results showed that each level of 25(OH)D did not show a significant difference (Table 6). In a cross-tabulation analysis, the Beck BDI-II scores were categorized as mild, moderate, and severe depression; the NRS-F scores were categorized as absence or presence of fatigue; and GSDS scores were categorized as absence or presence of sleep disorder. None of the results showed a significant difference in each level of 25(OH)D.

**Table 5:** Participant questionnaire score of three class shift nurses.

Variables	Shift			*p*
	Day (n = 34)	Evening (n = 30)	Night (n = 31)	
**BDI-II**				
Total score	10.5 (7.0–17.3)	12.5 (7.8–19.3)	16.0 (12.0–19.0)	0.245^b^
Normal	20 (58.8%)	16 (53.3%)	11 (35.5%)	0.572^c^
Mild depression	9 (26.5%)	7 (23.3%)	13 (41.9%)	
Moderate depression	3 (8.8%)	4 (13.3%)	4 (12.9%)	
Severe depression	2 (5.9%)	3 (10.0%)	3 (9.7%)	
**NRS-F**				
Total score	34.0 ± 13.0	37.9 ± 12.7	39.0 ± 15.6	0.302^a^
Average score	4.9 ± 1.9	5.4 ± 1.8	5.6 ± 2.2	0.302^a^
Fatigue	27 (33.8%)	26 (32.5%)	27 (33.8%)	0.643^c^
Non-fatigue	7 (46.7%)	4 (26.7%)	4 (26.7%)	
**GSDS**				
Total score	46.0 ± 12.8	46.4 ± 13.1	53.3 ± 12.5	0.043*^₤￥a^
Average score	3.1 ± 0.9	3.1 ± 0.9	3.6 ± 0.8	0.043*^₤￥a^
Sleep disorder	25 (33.3%)	23 (30.7%)	27 (36.0%)	0.38^d^
Non-sleep disorder	9 (45.0%)	7 (35.0%)	4 (20.0%)	

BDI-II, Beck Depression Inventory II; NRS-F, Numerical Rating Scale for Fatigue; GSDS, General Sleep Disturbance Scale.

*p-value < 0.05(^§^Day shift *v.s.* evening shift; ^₤^ evening shift *v.s.* night shift; ^￥^Day shift *v.s.* night shift). The power after calculation and analysis after the event: the number of subjects is 95 (n = 95), and the ANOVA test is carried out in three groups, and the calculated power is 0.6550386.

^a^Data were continuous and presented as mean ± SD using ANOVA, The Tukey–Kramer test was performed for the post hoc multiple of ANOVA.

^b^Data were continuous and presented as median (IQR) using Kruskal–Wallis test, The Dunn test was performed for the post hoc multiple of Kruskal–Wallis test.

^c^Data were categorical and presented as n (%) using Fisher's exact test.

^d^Data were categorical and presented as n (%) using chi-squared test.

**Table 6:** Participant questionnaire score according to 25(OH)D_3_ concentrations.

Variables	Serum 25(OH)D_3_, ng/ml			*p*
	< 20 (n = 46)	20–29 (n = 41)	≧ 30 (n = 8)	
**BDI-II**				
Total score	12.5 (7.0–19.0)	15.0 (8.5–19.0)	14.0 (9.0–31.3)	0.561^b^
Normal	26 (56.5%)	17 (41.5%)	4 (50%)	0.402^c^
Mild depression	11 (23.9%)	16 (39.0%)	2 (25.0%)	
Moderate depression	6 (13.0%)	5 (12.2%)	0 (0.0%)	
Severe depression	3 (6.5%)	3 (7.3%)	2 (25.0%)	
**NRS-F**				
Total score	38.4 ± 13.6	35.2 ± 13.8	36.3 ± 16.4	0.577^a^
Average score	5.5 ± 1.9	5.0 ± 2.0	5.2 ± 2.3	0.577^a^
Fatigue	42 (52.5%)	32 (40%)	6 (7.5%)	0.181^d^
Non-fatigue	4 (26.7%)	9 (60.0%)	2 (13.2%)	
**GSDS**				
Total score	50.0 ± 13.8	47.1 ± 12.5	46.9 ± 12.4	0.546^a^
Average score	3.3 ± 0.9	3.1 ± 0.8	3.1 ± 0.8	0.546^a^
Sleep disorder	38 (50.7%)	31 (41.3%)	6 (8.0%)	0.697^d^
Non-sleep disorder	8 (40.0%)	10 (50.0%)	2 (10.0%)	

BDI-II, Beck Depression Inventory II; NRS-F, Numerical Rating Scale for Fatigue; GSDS, General Sleep Disturbance Scale.

*p-value < 0.05. The power after calculation and analysis after the event: the number of subjects is 95 (n = 95), and the ANOVA test is carried out in three groups, and the calculated power is 0.6550386.

^a^Data were continuous and presented as mean ± SD using ANOVA.

^b^Data were continuous and presented as median (IQR) using Kruskal–Wallis test.

^c^Data were categorical and presented as n (%) using Fisher's exact test.

^d^Data were categorical and presented as n (%) using chi-squared test.

All data generated or analysed during this study are included in this published article.

## Discussion

According to the 2005–2008 National Nutrition Survey conducted in Taiwan, the average 25 (OH)D concentration of 2,596 adults aged 19 years and older was 18.1. In this study, the 25(OH)D levels of the three groups of shift nurses were as follows: 21.1 ± 6.2 ng/ml for day shifts, 21.3 ± 6.9 ng/ml for evening shifts, and 20.1 ± 4.7 ng/ml for night shifts; these results were like those of the National Health Survey. In Iran, Thailand, and India, studies conducted in nursing staff showed that 89%, 95.4%, and 94% of nurses had 25(OH)D deficiency (< 30 ng/ml)^31–33^. These findings are consistent with those of the present study. In another study conducted in female nurses in Iran, the serum 25(OH)D levels in young nurses (< 50 years) was lower than that in older nurses (> 50 years)^34^. The participants in this study were younger female nurses, and age possibly contributed to the lower 25(OH)D levels in this group.

The incidence of depression among nurses was 61.7%, and 74.9% of them had mild depression in China^7^. Our results showed that all three groups of nurses had mild depression. This study found no significant differences in the 25(OH)D levels and depression incidence, although many studies have shown an inverse relationship between 25(OH)D and depression, probably due to the small sample size. However, results of previous studies are consistent with the findings of this study. In the 2005–2006 United States National Health and Nutrition Survey, the results did not show a significant correlation between 25(OH)D levels and mild depression, moderate-to-severe depression, and severe depression in American adults^35^. The study conducted by Pan et al. also found that 25(OH)D level was not associated with depression incidence. They also pointed out that due to poor eating habits and lack of outdoor activities, the relationship between 25(OH)D and depression incidence could not be verified^36^. Eating habits and outdoor activities might also cause of 25(OH)D deficiency in this study possibly.

We showed that nurses experience a high degree of fatigue (average score: ≥ 3.3), but it is not related to level of 25(OH)D. Other study also pointed out that 37.1% of nurses experience fatigue in Taiwan^37^. In our study, the nurses with a 25(OH)D level of ≥ 30 ng/ml had a trend of lower fatigue score than the other two groups, but the difference was not significant. But there were only 8 subjects in this group, so it is difficult to present a significant difference. In a double-blind placebo-controlled clinical trial^38^, 120 patients with fatigue and 25(OH)D deficiency (serum 25(OH)D < 20 μg/l) were recruited and randomly assigned to receive an oral dose of 100,000 units of 25(OH)D or placebo. After 4 weeks, the fatigue of patients with 25(OH)D deficiency who received 25(OH)D supplementation significantly improved. However, the frequency of sun exposure and physical activity in each study population were not taken into account.

A previous study^39^ showed no difference in the degree of fatigue between night-shift and day-shift nurses; however, night-shift nurses had worse sleep quality than day-shift nurses, which is consistent with our study results; despite the presence of fatigue and sleep disturbance, the scores of night-shift nurses were significantly higher than those in the evening shift and day shift, but the level of fatigue of the three groups of nurses did not show a significant difference. Another study has pointed out that young nursing staff with 1–2 years of experience are more likely to have a shift work disorder^40^, also in our study. When comparing the night-shift and day-shift nurses, results showed that night-shift nurses are 20 years younger and have higher incidence of sleep disorders than day-shift nurses. A previous study examined the relationship between the daytime sleepiness (assessed using the Epworth sleepiness scale) and 25(OH)D concentration in white and black patients. No significant correlation was observed between daytime sleepiness and 25(OH)D in the white group^41^. In our study, also no significant difference was found between 25(OH)D levels and sleep disturbance after grouping the nurses according to 25(OH)D levels.

In inflammatory factors, in other study^42^, only the concentration of TNF-α in shift nurses was significantly higher than that in day-shift nurses, while IL-6 and IFN-γ were not significantly different. However, inflammatory factors were no significantly different in three groups in this study. A possible reason is the blood samples were not obtained at the end of night shift working time, which may have affected the results.

This study has several limitations. In the future, it may be possible to use objective testing methods, such as cortisol, to detect depression symptoms, and an exercise meter to record the quality of sleep. In addition, future studies should include participants of different ages and a larger sample size. Moreover, the number of samples with sufficient amounts of 25(OH)D (≥ 30 ng/dl) was relatively small; hence, it was difficult to judge a significant difference in the results. In addition, further research is needed to examine the relationship between sunlight and production of 25(OH)D in the body, as well as explore other factors that can effectively improve the depression symptoms including eating habits and lifestyle.

## Conclusions

In this study, we found that night-shift nurses had higher mental disorders and 91.6% of shift nurses have insufficient and severe levels of 25(OH)D, however mental disorders did not relate levels of 25(OH)D. In the future, a larger sample should be used to examine the level of depression, fatigue, and sleep disorders further objectively among shift nurses and explore their association with levels of 25(OH)D.

## Data Availability

All data generated or analysed during this study are included in this published article.
